# A health-promoting role of exclusive breastfeeding on infants through restoring delivery mode-induced gut microbiota perturbations

**DOI:** 10.3389/fmicb.2023.1163269

**Published:** 2023-07-10

**Authors:** Yu Liu, Jingmei Ma, Baoli Zhu, Fei Liu, Shengtang Qin, Na Lv, Ye Feng, Shuxian Wang, Huixia Yang

**Affiliations:** ^1^Department of Obstetrics and Gynecology, Peking University First Hospital, Beijing, China; ^2^Beijing Key Laboratory of Maternal Fetal Medicine of Gestational Diabetes Mellitus, Beijing, China; ^3^Key Laboratory of Pathogenic Microbiology and Immunology/Institute of Microbiology, Chinese Academy of Science, Beijing, China

**Keywords:** gut microbiota, early life, delivery mode, exclusive breastfeeding, disease susceptibility

## Abstract

The establishment of human gut microbiota in early life is closely associated with both short- and long-term infant health. Delivery mode and feeding pattern are two important determinants of infant gut microbiota. In this longitudinal cohort study, we examined the interplay between the delivery mode and feeding pattern on the dynamics of infant gut microbiota from 6 weeks to 6 months post-delivery in 139 infants. We also assessed the relationship between infant respiratory infection susceptibility and gut microbial changes associated with delivery mode and feeding pattern. At 6 weeks postpartum, the composition and structure of gut microbiota of cesarean section-delivered (CSD) infants differed from those of vaginally delivered (VD) infants, with decreased *Bacteroides* and *Escherichia-Shigella* and increased *Klebsiella, Veillonella*, and *Enterococcus*. At 6 months postpartum, these delivery mode-induced microbial shifts were restored by exclusive breastfeeding, resulting in similar gut microbial profiles between VD and CSD infants who were exclusively breastfed (*P* = 0.57) and more variable gut microbial profiles between VD and CSD infants who were mixed fed (*P* < 0.001). We identified that the VD-associated genera were enriched in healthy infants, while the CSD-associated genera were enriched in infants who suffered from respiratory infections. Our findings indicate that exclusive breastfeeding may play a health-promoting role by reducing infant respiratory infection susceptibility through the restoration of gut microbiota perturbations caused by cesarean section.

## Introduction

The gut microbiota is regarded as a complex and dynamic organ that interacts with the host metabolic pathways, immune responses, and developmental processes, influencing long-term host health (Tamburini et al., 2016; Robertson et al., 2019; Brodin, 2022). During the first years of life, the human gut microbiota develops toward an adult-like community by 2–3 years old, exhibiting a highly stage-specific progression (Stewart et al., 2018). The maturation of gut microbiota is shaped by numerous perinatal factors, including delivery mode (Reyman et al., 2019; Shao et al., 2019; Song et al., 2021), feeding pattern (Stewart et al., 2018; Fehr et al., 2020), antibiotic exposure (Bokulich et al., 2016), and gestational age (Fouhy et al., 2019). Recent longitudinal cohort studies have proven that delivery mode is a major determinant of gut microbiota during the first weeks of life, with cesarean section delivery (CSD) disrupting the natural transmission of gut microbiota from mothers to offspring (Shao et al., 2019). Specifically, the enrichment of *Bifidobacterium, Escherichia, Bacteroides*, and *Parabacteroides* in vaginally delivered (VD) infants can promote human milk oligosaccharide (HMO) utilization (Wang et al., 2019) and immune stimulation in early life (Jakobsson et al., 2014; Wampach et al., 2018). In contrast, the gut microbiota of infants delivered by cesarean section is dominated by *Enterococcus, Staphylococcus, Streptococcus*, and *Klebsiella*, which are associated with the hospital environment (Lax et al., 2017).

A growing body of evidence shows that cesarean section delivery is associated with adverse effects on infant and child immune development (Pattaroni et al., 2018), resulting in higher rates of asthma (Roduit et al., 2009; Tang et al., 2021) and respiratory infections (RIs) as morbidities (Bosch et al., 2016; Baumfeld et al., 2018; Reyman et al., 2019). A reduction in health-promoting *Bifidobacterium* and an enrichment in potentially pathogenic *Enterococcus* and *Klebsiella* in cesarean section-delivered infants have been linked with more RIs during the first year of life (Reyman et al., 2019), indicating a mediating role of gut microbial changes in the cesarean delivery-induced disease susceptibility.

The effect of the delivery mode gradually diminishes by 6 months of life (Hill et al., 2017) or earlier (Reyman et al., 2019), indicating that the gut microbiota could recover from the state associated with cesarean section delivery. Our previous study demonstrated that shifts in infant gut microbiota associated with cesarean section delivery were alleviated by exclusive breastfeeding (EBF) in the first several weeks of life, suggesting an interactive impact on feeding pattern and the delivery mode of gut microbiota (Liu et al., 2019). Additionally, prolonged breastfeeding duration influenced the gut microbiota of CSD infants but not VD infants at 24 weeks of age, indicating that CSD infants may benefit from breastmilk by obtaining specific bacteria, initially lacking due to delivery mode (Hill et al., 2017). The gut microbiota of exclusively breastfed and formula-fed infants remain distinct (Backhed et al., 2015b), even when specific components are added to the formula to promote breastfed-like microbial communities (Baumann-Dudenhoeffer et al., 2018), demonstrating that the nutritional and immune benefits of breastfeeding were indispensable (Gopalakrishna and Hand, 2020; Gridneva et al., 2021; Donald et al., 2022).

However, few studies have explored the relationship between infant respiratory health and gut microbiota shifts associated with delivery mode and feeding pattern. In this prospective cohort study containing 139 infants, we investigated the health-promoting role of exclusive breastfeeding on infant gut microbiota shifts induced by cesarean section, which were associated with reduced respiratory infection susceptibility during the first months of life.

## Materials and methods

### Ethics

Written informed consent was obtained from the legal guardian for the publication of any potentially identifiable data included in this article.

### Study population

This ongoing prospective cohort study has been conducted at Peking University First Hospital since October 2017, which recruited 139 infants at 6 weeks postpartum, and 72.7% (101/139) of them completed the 6-month follow-up. This study was approved by the Institutional Ethics Committee of Peking University First Hospital (V2.0/201504.20), and all the participants or legal guardians provided written informed consent.

### Clinical information

Prenatal and perinatal information was obtained from electronic medical records and questionnaire surveys (Supplementary Table 1), including maternal age, gravidity, parity, pre-pregnancy body mass index (BMI), delivery mode, gestational age, infant sex, birth weight, feeding patterns, and the occurrence of respiratory infection (RI) events during the first 6 months of age. Considering the confounding effect of delivery mode, we also recorded the type (labored or elective) and cause of cesarean section. EBF is defined as feeding with breast milk exclusively after birth. MF is defined as feeding with a mixture of varying proportions of breast milk and formula milk. RI events are defined as the occurrence of the following mother-reported symptoms: pneumonia, bronchitis, or fever (>38°C) accompanied by snuffling, sneezing, coughing, or wheezing.

### Sample collection, DNA extraction, and 16s rRNA gene sequencing

A total of 139 infants were collected at 6 weeks, with 101 infants longitudinally collected at 6 months. Fresh stool samples were self-collected at home, according to the standardized protocol, as described in a previous study (Liu et al., 2019). All stool samples were frozen at −80°C within 2 h. DNA was extracted with the QIAamp PowerFecal DNA Kit (Qiagen, Hilden, Germany), following the manufacturer's protocols. The V3-V4 region of the 16S rRNA gene was amplified by polymerase chain reaction (PCR) with 341 forward primers (5′ CCTACGGGNBGCASCAG) and 805 reverse primers (5′ GACTACNVGGGTATCTAATCC). PCR was performed in a 25-μl volume with 1 U of HiFi HotStart DNA Polymerase (KK2502, Kapa Biosystems, Cape Town, South Africa), 12.5 ng of template DNA, and 5 μM of forward and reverse primers with the following amplification program: initial denaturation at 95°C for 3 min; 25 cycles of denaturation at 95°C for 30 s, annealing at 55°C for 30 s, and extension at 72°C for 30 s; and final extension at 72°C for 5 min and hold at 4°C. AMPure XP beads (A63882, Beckman) were used to purify the 16S V3-V4 amplicons away from the free primers and primer dimers.

For Illumina sequencing adapter attachment, PCR was performed for a second time with Illumina sequencing primers under the same conditions as the first time, only with seven cycles and an annealing step increased to 1 min. DNA libraries were quantified with a NanoDrop 2000 system and purified with AMPure XP beads before sequencing on the Illumina HiSeq 2500 platform. Fast Length Adjustment of Short Reads (FLASH) was used to merge paired-end reads from sequencing (Magoc and Salzberg, 2011). Low-quality reads were filtered with the FASTQ quality filter (-p 90 -q 25 -Q33) using the FASTX Toolkit 0.0.14, and chimeric reads were removed by USEARCH 64-bit v8.0.1517. The number of reads for each sample was normalized based on the smallest sample size by random subtraction.

### 16s rRNA data processing and statistical analyses

The 16s rRNA sequencing data were processed by Quantitative Insights Into Microbial Ecology (QIIME). Operational taxonomic units (OTUs) were aligned by the UCLUST algorithm with 97% identity and taxonomically classified using the SILVA 16S rRNA database v128. The bacterial compositions were visualized with a heatmap, which was generated via Seaborn, a Python data visualization library. Alpha diversity was evaluated by Shannon and Simpson indexes. Beta diversity was calculated based on weighted UniFrac and Bray–Curtis distance matrices and visualized by principal coordinates analysis (PCoA) of weighted UniFrac and Bray–Curtis distance matrices, in 1,000 permutations, and statistical comparisons of groups were calculated by multivariate permutational analysis of variance (PERMANOVA) methods using the Adonis function in the R package “vegan.” Metric variables are shown as the mean ± SD or median (interquartile range) and compared with Student's *t-*test or Mann–Whitney *U*-test, according to the normality of the data distribution. The Chi-squared and Fisher's exact tests were used to compare the proportions of analyses. The significance was set at a *P*-value of < 0.05. GraphPad Prism version 7.0 (GraphPad Software, San Diego, CA) was used for statistical and graphical preparation.

## Results

### Population characteristics

In this longitudinal study, stool samples were collected from each individual at ~6 weeks postpartum (6W, *n* = 139) and 6 months postpartum (6M, *n* = 101), and some of the individuals had been included in our previous study (Liu et al., 2019). Among 139 infants, 93 (66.9%) infants were born by vaginal delivery, and 46 infants were born by cesarean section delivery. Among 101 infants, 71 (70.1%) infants were born by vaginal delivery, and 30 infants were born by cesarean section delivery. Basic characteristics at 6W postpartum and 6M postpartum stratified by delivery mode, are shown in Table 1. Clinical variables were similar between the VD and CSD groups, with the exception of gestational age (*P* < 0.001 at 6W; *P* = 0.03 at 6M), which was intrinsically related to the delivery mode. Notably, VD infants were more likely to receive exclusive breastfeeding; this difference persisted to 6M, although significance was not reached. The occurrence rate of RI over the first 6 months was significantly higher in CSD infants than in VD infants (*P* = 0.02).

**Table 1:** Infants' clinical parameters stratified by delivery mode at 6W and 6M.

**Characteristics**	**6W**			**6M**		
	**Mean** ±**SD, median (IQR) or** ***n*** **(%)**			**Mean** ±**SD, median (IQR) or** ***n*** **(%)**		
**VD (*N* = 93)**	**CSD (*N* = 46)**	***P*-value**	**VD (*N* = 71)**	**CSD (*N* = 30)**	***P*-value**
Maternal age (years)	31.67 ± 3.77	32.72 ± 3.87	0.10	31.41 ± 3.85	32.90 ± 4.29	0.08
Gestational age (weeks)	39.22 ± 1.26	38.41 ± 1.24	*** < 0.001***	39.14 ± 1.25	38.7 ± 1.09	***0.03***
Maternal pre-pregnancy BMI	22.19 ± 3.27	23.19 ± 3.12	0.08	22.08 ± 3.11	23.37 ± 3.25	0.06
Gravity	2 (1, 2)	2 (1, 3)	0.09	1 (1, 2)	2 (1, 3)	0.11
Parity	1 (1, 2)	1 (1, 2)	0.19	1 (1, 1)	1 (1, 2)	0.29
Sampling time (days)	48 (44, 55)	49 (43.75, 58.75)	0.48	208 (199, 219)	202 (187, 217)	0.15
Birth weight (g)	3,302 ± 382.2	3,296 ± 469.5	0.94	3,280 ± 366.3	3,377 ± 468.9	0.27
Gender			0.15			0.19
Male	54 (58.06)	20 (43.48)		42 (59.15)	13 (43.33)	
Female	39 (41.94)	26 (56.52)		29 (40.85)	17 (56.67)	
Feeding patterns			0.06			0.1
EBF	68 (73.12)	26 (56.52)		27 (38.03)	6 (20)	
MF	25 (26.88)	20 (43.48)		44 (61.97)	24 (80)	
RIs	-	-		9 (12.68)	10 (33.33)	***0.02***

Clinical characteristics stratified by delivery mode. Continuous variables are shown as mean ± SD (normally distributed) or median with IQR (non-normally distributed); categorical variables are shown in absolute numbers with percentages (%). The *P*-value of variables is determined by Student's *t*-test, the Mann–Whitney *U*-test, or Fisher's exact test, and the significance is in bold and italicized.

6W, 6 weeks postpartum; 6M, 6 months postpartum; VD, vaginally delivered; CSD, cesarean section delivered; SD, standard deviation; IQR, interquartile range; EBF, exclusive breastfeeding; MF, mixed feeding; RIs, respiratory infections.

### Infant gut microbiota clusters according to age

In total, 38,955,382 bacterial reads were identified in 240 fecal samples, which were annotated into 654 OTUs distributed over 8 bacterial phyla. Firmicutes (43.9%) was the most abundant phylum, followed by Actinobacteria (21.9%), Proteobacteria (21.2%), and Bacteroidetes (12.6%).

**Figure 1:**
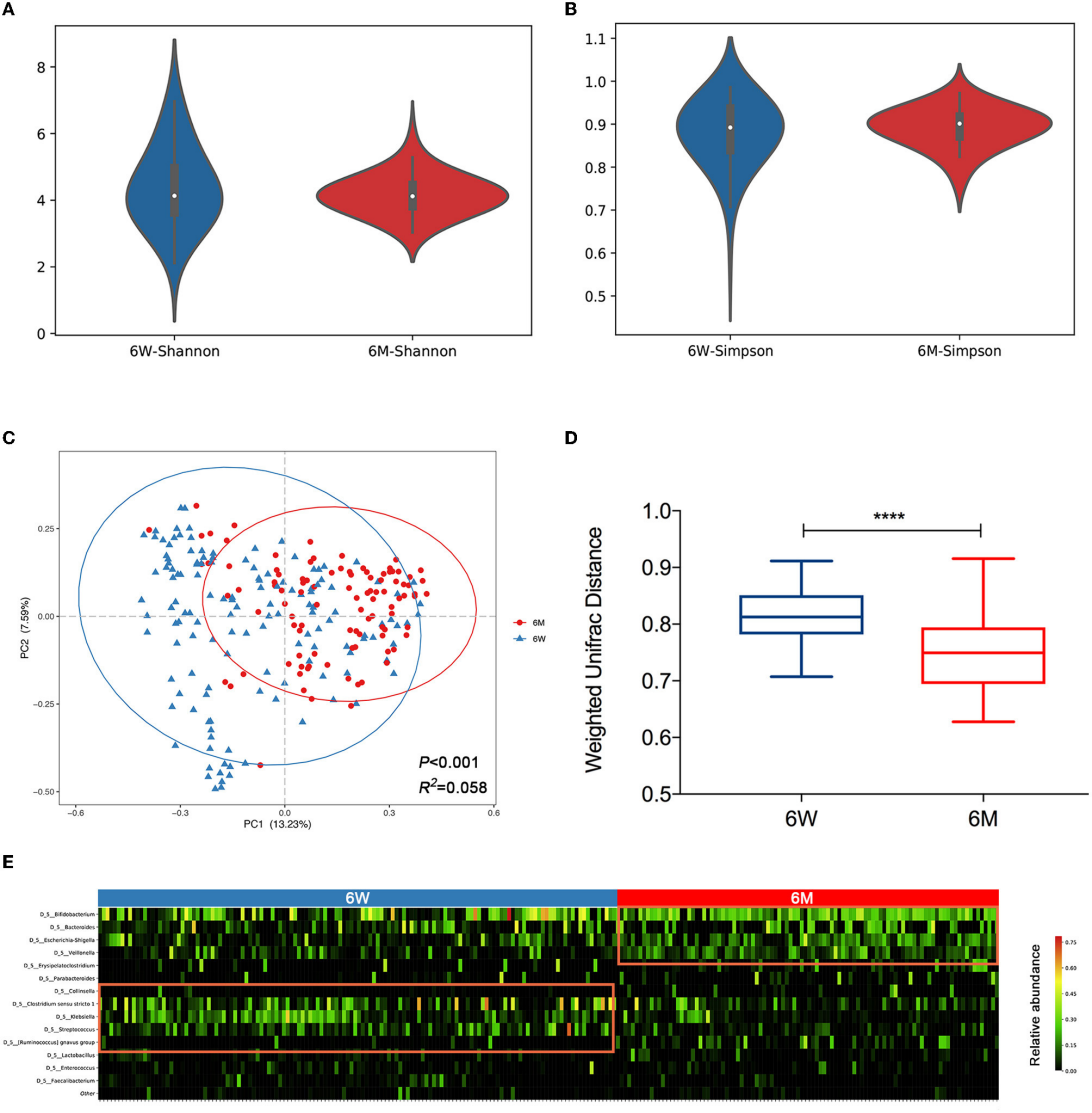
The distinct composition and community structure of gut microbiota in 6-week-old and 6-month-old infants. Alpha diversity is shown as the Shannon index **(A)** and Simpson index **(B)**. **(C)** Principal coordinates analysis (PCoA) based on Bray–Curtis distance is shown along the first two principal coordinate (PC) axes with Adonis *p*-value and effect sizes (*R*^2^). **(D)** Comparison of the weighted UniFrac within-group distance of infants. The shorter distance indicated greater similarity in microbial community composition. Boxplots were shown as medians with range. **(E)** A heatmap of the top 15 bacterial OTUs (average relative abundance >1%) clustered according to age. Values range from low (black) to high (red). A significant difference was determined by Student's *t-*test (^****^p < 0.0001). 6W, 6-weeks-old infants; 6M, 6-months-old infants.

We first conducted a chronological comparison of infant gut microbial composition and community structure between 6 weeks and 6 months of age. No significant difference was found in alpha diversity between the 6W and 6M groups, according to either the Shannon index (Figure 1A, *P* = 0.41) or the Simpson index (Figure 1B, *P* = 0.43). Beta diversity was assessed using Bray–Curtis distance matrices, and infant gut microbiota structure remained markedly distinct between 6W and 6M (Figure 1C, Adonis, *P* < 0.001, *R*^2^ = 0.058). The gut microbiota of infants converged toward a tighter cluster at 6M compared to 6W. This observation was further consolidated by a comparison of the weighted UniFrac within-group distance (Figure 1D, *P* < 0.0001), suggesting that infants shared a more homogeneous microbial community with aged.

A heatmap of the top 15 genera based on an average relative abundance of >1% was used to identify age-associated patterns in infant gut microbiota. The heatmap revealed that *Clostridium sensu stricto1, Klebsiella*, and *Streptococcus* (highlighted on the left side of the heatmap), which are associated with the hospital environment (Lax et al., 2017), are present at higher relative abundances at 6W (*P* < 0.05, *P* < 0.001, and *P* < 0.001, respectively). In contrast, *Bifidobacterium, Bacteroides, Escherichia-Shigella*, and *Veillonella* (highlighted on the right side of the heatmap) were enriched at 6M (*P* < 0.05, *P* = 0.11, *P* < 0.001, and *P* < 0.001, respectively, Figure 1E).

### The effect of delivery mode on infant gut microbiota dissipates with age in a feeding pattern-dependent manner

**Figure 2:**
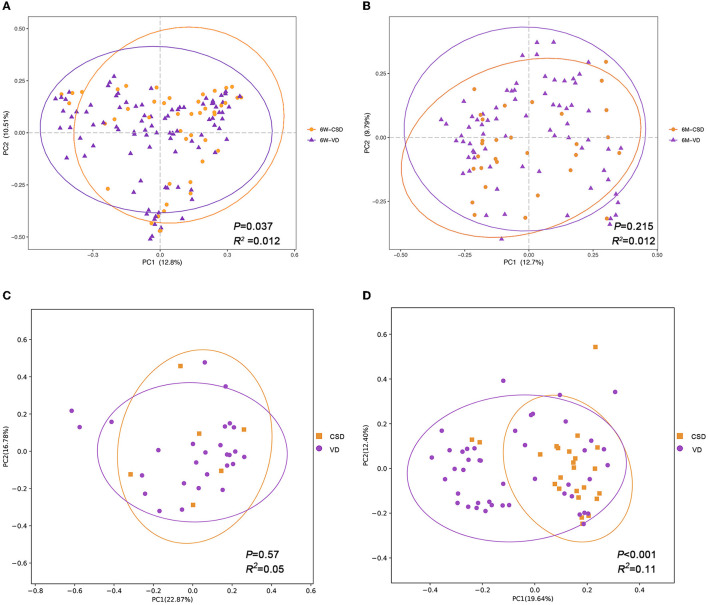
The impact of delivery mode on infant gut microbiota is gradually diminished and influenced by exclusive breastfeeding. Principal coordinates analysis (PCoA) plots of infant samples stratified according to the delivery mode, based on Bray–Curtis distance, are shown along the first two principal coordinate (PC) axes with Adonis *p*-value and effect sizes (*R*^2^). **(A)** Infants aged 6 weeks. **(B)** Infants aged 6 months. **(C)** Infants who were exclusively breastfed during 6 months. **(D)** Infants who were mixed fed during 6 months. VD, vaginally delivered; CSD, cesarean section delivered.

We further investigated the impact of delivery mode on the infant gut microbiota, stratified by age. At 6W, a significant difference was found in the gut microbial community structure between VD and CSD infants (Figure 2A, Adonis, *P* = 0.037, *R*^2^ = 0.012). However, this difference dissipated at 6M (Figure 2B, Adonis, *P* = 0.215, *R*^2^ = 0.012). These results are consistent with previous studies (Stewart et al., 2018; Wampach et al., 2018; Reyman et al., 2019; Shao et al., 2019), demonstrating that delivery mode is a major determinant of gut microbiota in early life but with a gradually diminished effect throughout infancy.

Considering the interplay between breastfeeding and delivery mode in relation to the infant gut microbiota (Hill et al., 2017; Liu et al., 2019), we hypothesized that the impact of delivery mode would be influenced by feeding patterns. To test this hypothesis, we conducted a stratified analysis based on samples collected at 6M separately (*n* = 101). The 101 infants were divided into two groups according to feeding patterns: exclusive breastfeeding (*n* = 33) and mixed feeding (*n* = 68) groups. As expected, no significant difference in the gut microbiota structure was observed between VD (*n* = 27) and CSD (*n* = 6) infants in the breastfeeding groups (Figure 2C, Adonis, *P* = 0.57, *R*^2^ = 0.05). However, in the mixed feeding groups, a stronger significant difference in gut microbiota structure was observed between VD (*n* = 44) and CSD (*n* = 24) infants, with a higher *R*^2^ value of 0.11 and a *P*-value of <0.001 (Figure 2D). This suggested that breastfeeding may alleviate the disturbance of gut microbiota in CSD infants.

**Figure 3:**
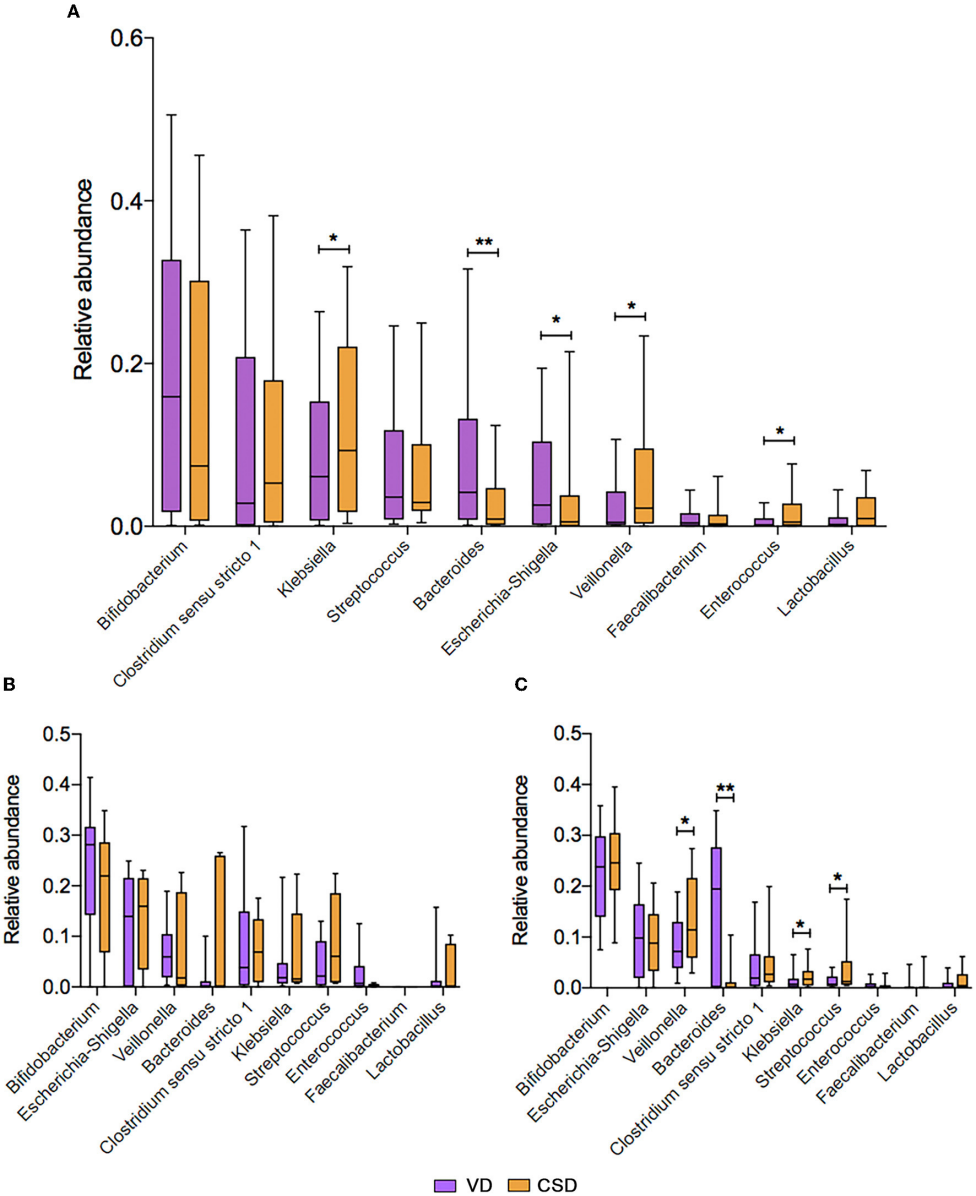
The mean relative abundance of the top 10 genera associated with the delivery mode. **(A)** The comparison of mean relative abundance of the top 10 genera between VD and CSD infants at P6W. The comparison of mean relative abundance of the top 10 genera between VD and CSD infants who were exclusively breastfed **(B)** and mixed fed **(C)** at P6M. A significant difference was determined by Student's *t-*test or the Mann–Whitney *U*-test (^*^p < 0.05; ^**^p < 0.01). VD, vaginally delivered; CSD, cesarean section delivered.

Next, we aimed to identify specific taxa responsible for this feeding pattern-dependent change. The average relative abundances of the top 10 genera were compared between VD and CSD infants via a cross-sectional analysis. At 6W, all VD infants (*n* = 93) were enriched in *Bacteroides* and *Escherichia-Shigella* (Figure 3A, *P* < 0.01 and *P* < 0.05, respectively), while CSD infants (*n* = 46) were depleted of these two commensal genera and enriched in *Klebsiella, Veillonella*, and *Enterococcus* (Figure 3A, *P* < 0.05 for all). These findings are in agreement with recent observations in other cohort studies (Backhed et al., 2015a; Reyman et al., 2019; Shao et al., 2019). At 6M, the relative abundances of the top 10 genera were comparable between VD and CSD infants in the breastfeeding groups (Figure 3B), whereas the relative abundances of the aforementioned discriminative taxa remained different between VD and CSD infants in the mixed-feeding groups, with a lower relative abundance of *Bacteroides* (Figure 3C, *P* < 0.01) and a higher abundance of *Klebsiella, Veillonella*, and *Streptococcus* in CSD infants (*P* < 0.05 for all), while the relative abundances of *Escherichia-Shigella* were comparable between VD and CSD infants (*P* = 0.6).

### Impact of delivery mode-induced microbiota changes on infant health

**Figure 4:**
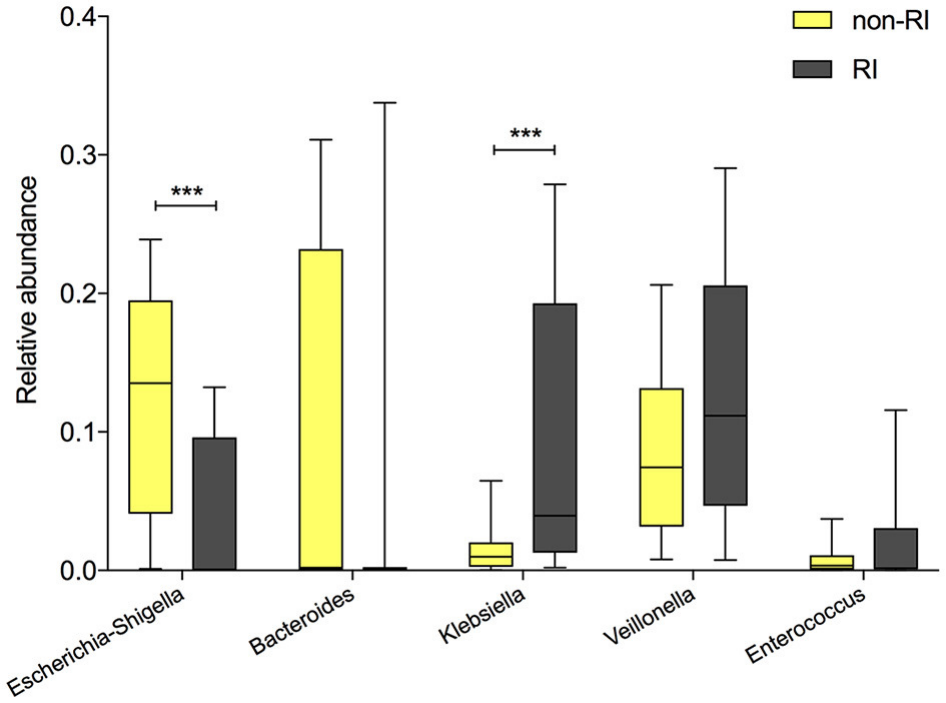
The comparison of the relative abundance of delivery mode-associated bacterial taxa between the RI and non-RI groups. A significant difference was determined by Student's *t-*test or the Mann–Whitney *U*-test (^***^p < 0.001). RI, respiratory infection.

Since previous studies have reported an association between delivery mode and susceptibility to respiratory diseases (Baumfeld et al., 2018), which might be mediated by gut microbiota composition (Reyman et al., 2019), we further investigated the relationship between delivery mode and infant RI status, according to parental self-reporting over the first 6 months. Indeed, the incidence of RI was significantly higher in CSD infants (9/71) than in VD infants (10/30, 12.7 vs. 33.3%, *P* = 0.02), prompting our hypothesis that delivery mode-induced gut microbial changes are associated with the RI status. Consequently, all infants at 6 months postpartum (*n* = 101) were divided into two groups based on the occurrence of at least one RI event: the RI group (*n* = 19) and the non-RI group (*n* = 82). The relative abundances of the five delivery mode-associated bacterial taxa (*Bacteroides, Escherichia-Shigella, Klebsiella, Veillonella*, and *Enterococcus*) were compared between the RI and non-RI groups. As expected, the VD-associated *Escherichia-Shigella* was more abundant in the non-RI group (Figure 4, *P* < 0.0001), whereas CSD-associated *Klebsiella* was enriched in the RI group (*P* < 0.001). Additionally, *Bacteroides*, enriched in VD infants, was more abundant in the non-RI group (*P* = 0.32), while *Veillonella* and *Enterococcus*, enriched in CSD infants, were more abundant in the RI group (*P* = 0.15, *P* = 0.78, respectively), although the difference did not reach the statistical significance.

Considering the mediating role of the feeding pattern on the relationship between delivery mode and gut microbiota, we further categorized infants (*n* = 101) into four groups based on the combination of delivery mode and feeding pattern: vaginally delivered and exclusively breastfed (VB, *n* = 27), vaginally delivered and mixed fed (VM, *n* = 44), cesarean delivered and exclusively breastfed (CB, *n* = 6), and cesarean delivered and mixed fed (CM, *n* = 24). We defined the VB group as the healthy reference group and found that the incidence of RIs of the CB group (1/6, 16.7%) was similar to that of the VB group (5/27, 18.5%), which was much lower than that of the CM group (9/24, 36%). Although the difference did not reach statistical significance (*P* = 0.26), this result indicated that exclusive breastfeeding may play a health-promoting role by rectifying the gut microbial composition induced by the cesarean section delivery.

## Discussion

Gut microbiota establishment in early life is essential to host health and disease susceptibility, which is greatly influenced by two perinatal factors, delivery mode and feeding pattern. However, the knowledge of the relationship between infant health and gut microbiota shifts associated with delivery mode and feeding pattern is limited. In this longitudinal study of 139 infants, we observed that the perturbations of infant gut microbiota caused by cesarean section were associated with higher risks of respiratory infection in the first months of life. These delivery mode-induced microbiota shifts were alleviated by exclusive breastfeeding, resulting in reduced respiratory infection susceptibility. Our study indicates a health-promoting role of exclusive breastfeeding on infants through restoring the delivery mode-induced gut microbial perturbations.

In recent decades, the rates of cesarean section have steadily increased worldwide, reaching 32.4% in the US and 34.9% in China (Li et al., 2017). This rise has been linked to a higher risk of metabolic and immune disorders in CSD offspring in the short and long terms (Huh et al., 2012; Sevelsted et al., 2015). Although the exact mechanism remains poorly understood, increasing evidence has demonstrated that gut microbiota might mediate the association between cesarean section delivery and susceptibility to obesity (Tun et al., 2018), diabetes mellitus (Andersen et al., 2020; Chavarro et al., 2020), asthma (Stokholm et al., 2020), respiratory tract infections (Reyman et al., 2019), and chronic immune issues (Wampach et al., 2018). Consistent with these findings, we observed a higher incidence of RIs during the first 6 months of life in CSD infants compared with VD infants. This outcome was related to low *Escherichia-Shigella* and high *Klebsiella* profiles in the intestine caused by cesarean section delivery. In addition to these two bacterial taxa, a decreased relative abundance of *Bacteroides* and the enrichment of *Veillonella* and *Enterococcus* were observed in our CSD infants, which aligns with previous studies (Reyman et al., 2019; Shao et al., 2019).

The lack of specific bacterial taxa in CSD infants has been shown to disturb the maturation of the infant intestine and immune systems (Jakobsson et al., 2014; Tamburini et al., 2016). For example, the low *Bacteroides* profile in CSD infants, considering a signature of disturbed gut microbiota development in early life (Shao et al., 2019), is related to a reduction in lipopolysaccharide (LPS) exposure, thus decreasing the stimulation of primary human immune cells and exerting a long-term impact on immune-mediated diseases (Jenmalm, 2011; Jakobsson et al., 2014; Wampach et al., 2018). *Klebsiella*, regarded as an opportunistic pathogen, is a common cause of hospital infection, and an increased ratio of *Klebsiella*/*Bifidobacterium* in early life is related to the latter development of pediatric allergies (Low et al., 2017). Combining our observation, the enrichment of *Klebsiella* may be involved not only in the risk of immunological disorders but also in the risk of infectious diseases in CSD offspring.

Consistent with previous studies (Hill et al., 2017; Reyman et al., 2019; Shao et al., 2019), we found that the effect of delivery mode on microbial profiles gradually decreased with infant age. Unique to our study, we further determined whether this dynamic trajectory was affected by feeding patterns, which was prompted by our previous observation that microbial alterations caused by cesarean section delivery could be rectified by EBF in a single cross-sectional analysis at 6 weeks of life (Liu et al., 2019). As expected, the dynamic effect of delivery mode on infant gut microbiota was dependent on the feeding pattern, demonstrating that the microbial compositional disturbance associated with cesarean section delivery could be corrected by EBF through increasing or decreasing specific bacterial taxa but not by MF. Taking *Klebsiella* as an example, the difference in relative abundance between VD and CSD infants at 6 weeks was rectified by EBF lasting for 6 months of life but exacerbated by MF.

A recent systematic review assessed that the relative abundances of particular gut-commensal bacteria genera were associated with childhood respiratory infection (Alcazar et al., 2022), suggesting that gut microbiota might be a determinant of childhood respiratory disease. In this study, we further compared the incidence of RIs among VB, CB, and CM infants, taking VB infants as a healthy reference. Although no significant difference was found, we observed a trend toward a higher incidence of RIs in CM infants than in CB infants. In terms of gut microbiota, our results provide evidence to support the previous viewpoint that prolonged EBF reduces the risk of infectious diseases in infancy (Duijts et al., 2010; Brodin, 2022), especially in CSD infants. However, the proportion and duration of EBF were significantly lower in CSD infants (Vestermark et al., 1991; Dewey et al., 2003).

Breast milk and its components exert an important influence on the nature of early-life immune responses during microbial colonization. Although artificial oligosaccharides are added to infant formula milk to mimic the composition of human breast milk, a significant difference in gut microbial composition is still observed between EBF and MF infants (Azad et al., 2013; Stewart et al., 2018). Presumably, IgA antibodies and maternal antibodies in breast milk help tip the balance toward tolerance and immune-microbe mutualism while providing passive immunity to defend against invasive bacteria (Brodin, 2022). In addition to the different components between breast milk and formula milk, the breast milk microbiota also plays an important role (Le Doare et al., 2018). The abundance of specific microbial strains of *Bifidobacterium, Lactobacillus, Enterococcus*, and *Staphylococcus* species in the infant's intestine increased with the proportion of daily breast milk intake in a dose-dependent manner (Pannaraj et al., 2017), strongly indicating the transfer of microbes from breast milk to the infant intestine (Martin et al., 2012). Furthermore, HMOs in breast milk are metabolized by microbiota, leading to the production of metabolites such as indole-3-lactic acid, which likely mediates some of the beneficial effects of breast milk early in life (Henrick et al., 2021). Taken together, these results highlight the importance of EBF, especially for CSD infants, and provide a potential reference for optimizing formula milk by adding specific bacterial taxa, resulting in a gut microbial profile resembling that of EBF infants.

The strengths of our study included the longitudinal sampling, which enabled us to assess the dynamic effect of delivery mode on infant gut microbiota. In addition, a mixed feeding regimen was included in our study, which was often introduced when mothers return to work or prepare to wean in China, while most previous studies usually focused on the comparison of gut microbiota in EBF and exclusively formula-fed infants (Azad et al., 2013; Gomez-Llorente et al., 2013; Bergstrom et al., 2014). Finally, we highlighted the association between infant clinical consequences and delivery mode-induced gut microbial perturbations. However, our study has some limitations. The incidence of respiratory infection was determined by the self-report of the mother, which may be biased by factitious factors. To investigate the relationship between health outcomes and delivery mode, detailed information about clinical phenotype was lacking.

## Conclusion

In conclusion, we assessed the dynamic impact of delivery mode on infant gut microbiota and attenuated it in a feeding pattern-dependent manner during the first months of life. The perturbation of infant gut microbiota caused by cesarean section was restored by exclusive breastfeeding, resulting in reduced respiratory infection susceptibility in the first years of life. Our study suggests a health-promoting role of exclusive breastfeeding on infants by restoring the delivery mode-induced gut microbial perturbations.

## Data availability statement

The datasets presented in this study can be found in online repositories. The names of the repository/repositories and accession number(s) can be found in the article/Supplementary material.

## Ethics statement

The studies involving human participants were reviewed and approved by Peking University First Hospital (V2.0/201504.20). Written informed consent to participate in this study was provided by the participants' legal guardian/next of kin.

## Author contributions

YL, JM, and HY conceived the study design. YL, SQ, YF, and SW were responsible for the recruitment and collection of samples. NL, FL, and BZ were responsible for the laboratory assays. YL performed the data analysis and completed the initial manuscript. HY revised the manuscript. All authors have read and approved the final version of the manuscript.
